# Differential susceptibility of PCR reactions to inhibitors: an important and unrecognised phenomenon

**DOI:** 10.1186/1756-0500-1-70

**Published:** 2008-08-28

**Authors:** Jim F Huggett, Tanya Novak, Jeremy A Garson, Clare Green, Stephen D Morris-Jones, Robert F Miller, Alimuddin Zumla

**Affiliations:** 1 Centre for Infectious Diseases and International Health, Windeyer Institute for Medical Sciences, 46 Cleveland Street, University College London, London, W1T 4JF, UK; 2 Centre for Virology, Windeyer Institute for Medical Sciences, University College London, London, W1T 4JF, UK; 3 Department of Microbiology, University College London Hospitals NHS Foundation Trust, Windeyer Institute for Medical Sciences, London, W1T 4JF, UK; 4 Research Department of Infection and Population Health, Division of Population Health, University College London, London, WC1E 6JB, UK

## Abstract

**Background:**

PCR inhibition by nucleic acid extracts is a well known yet poorly described phenomenon. Inhibition assessment generally depends on the assumption that inhibitors affect all PCR reactions to the same extent; i.e. that the reaction of interest and the control reaction are equally susceptible to inhibition. To test this assumption we performed inhibition assessment on DNA extracts from human urine samples, fresh urine and EDTA using different PCR reactions.

**Results:**

When copurified inhibitors were assessed using two different PCR reactions one reaction appeared to be inhibited whilst the other was not. Further experiments using various concentrations of unextracted urine to inhibit six different PCR reactions revealed that susceptibility to inhibition was highly variable between reactions. Similar results were obtained using EDTA as the PCR inhibitor. We could find no obvious explanation why one reaction should be more susceptible to inhibition than another, although a possible association with amplicon GC content was noted.

**Conclusion:**

These findings have serious implications for all PCR-based gene expression studies, including the relatively new PCR array method, and for both qualitative and quantitative PCR-based molecular diagnostic assays, suggesting that careful consideration should be given to inhibition compatibility when conducting PCR analyses. We have demonstrated unequivocally that it is not safe to assume that different PCR reactions are equally susceptible to inhibition by substances co-purified in nucleic acid extracts.

## Background

It is well known that the polymerase chain reaction (PCR) is susceptible to inhibitors [1-4] and many publications describe methods for assessing inhibition using spiked alien molecules of various types [5-9]. Inhibition in real-time PCR can be measured as the increase in threshold cycle (Ct) or crossing point (Cp) relative to an uninhibited control [10]. The presence of inhibitors has the potential to increase error, reduce assay resolution, and produce false results in both quantitative and qualitative PCR assays. Direct assessment of inhibition is not usually performed [1], but as real-time PCR analyses frequently include additional reactions to control for sample variation (normalisation) by measuring reference 'housekeeping' transcripts [11] or genomic DNA [12], an assessment of, and compensation for, inhibition is often conducted indirectly.

Using a spiked alien molecule (as an internal positive control) or reference gene to assess inhibition relies on the fundamental assumption that any inhibitor present within the sample will have an equal effect on both PCR reactions. However, there appears to be no evidence in the literature to substantiate this assumption. Intuitively, there is no fundamental reason why this assumption should be valid, and yet it underpins a significant proportion of the PCR analyses performed daily in research and diagnostic laboratories throughout the world. In this study we examine, using a model system, how a range of different reactions may be differentially affected by PCR inhibitors and discuss the implications of the unexpected findings.

## Methods

For more detailed methods please refer to the additional file.

### Urine donors

Fresh mid stream urine specimens were collected from 19 healthy adult volunteers. An aliquot from each specimen was cultured to exclude the presence of bacterial infection. Written informed consent was obtained from all participants and the appropriate hospital ethics committee approved the study.

### DNA extraction from urine

DNA was extracted from 10 ml urine using a protocol combining Q-sepharose™ Fast Flow (GE Healthcare Life Sciences, Buckinghamshire, UK) and a Viral RNA Mini Kit (Qiagen, Crawley, UK). DNA was eluted in 50 μl water and 5 μl of this used for the respective PCR reactions.

### Real-time PCR

Six real-time PCR reactions were used in this study as detailed in Tables 1 and 2. The SPUD [8], *Pj*HSP70a [12] and IS1081 [13] reactions have been previously described. All reactions were conducted in 12.5 μl volumes using QuantiTect Probe PCR kit 2 × master mix (Qiagen, Crawley, UK) and a Rotorgene 6000 thermocycler (Corbett Research, Cambridge, UK). PCR efficiencies were estimated using ten fold dilution series according to the formula E = 10^(-1/slope)^-1 [14]. Amplification curves were also assessed to establish what effect potential inhibitors had on gradient and endpoint fluorescence.

**Table 1:** Primer and probe sequences

Reaction	Oligoδ	Sequence
*Pj*HSP70a	F	CGTCTTGTAAACCACTTCATTGC
	R	AGTCCGTTTAGCACGCTCAC
	P	HEX 5' AAGAAAGATCTTTCAGGG 3' BHQ1*
mtLSU133	F	GCACTGAATATCTCGAGGGA
	R	ACTGTTCTGGGCTGTTTCC
	P	HEX 5' CTTATCGCACATAGTCTGATT 3'BHQ1*
CFP32	F	AGAAGCGAATACAGGCAAGG
	R	CGGACTGATCGGTGGTCT
	P	HEX 5' CGCCGAACTGGGTCGACCTTC 3' BHQ1
IS1081	F	CTGCTCTCGACGTTCATCGCCG
	R	GGCACGGGTGTCGAAATCACG
	P	HEX 5' ATTGGACCGCTCATCGCTGCGTTCGC 3' BHQ1
16S MTb	F	CAAGTCGAACGGAAAGGTCT
	R	GCAGATCACCCACGTGTTAC
	P	HEX 5' CCCGTTCGCCACTCGAGTATCTC 3' BHQ1
SPUD	F	AACTTGGCTTTAATGGACCTCCA
	R	ACATTCATCCTTACATGGCACCA
	P	FAM 5'TGCACAAGCTATGGAACACCACGT 3' BHQ1

n.b. ^δ ^Oligos; F: forward primer, R: reverse primer, P: hydrolysis probe.

* underline denotes locked nucleic acid (LNA) moieties

**Table 2:** PCR reaction parameters

					Reaction parameters		
Assay	F & R [Primer]	[Probe]	Annealing temp	95°C	Anneal	72°C	Wavelength excite/acquire
*Pj*HSP70a	600 nM	200 nM	60°C	10 sec	10 sec	20 sec	530 nm/555 nm
mtLSU133	700 nM	100 nM	56°C	10 sec	20 sec	20 sec	530 nm/555 nm
CFP32	600 nM	75 nM	60°C	10 sec	20 sec	16 sec	530 nm/555 nm
IS1081	600 nM	75 nM	60°C	10 sec	20 sec	16 sec	530 nm/555 nm
16S MTb	600 nM	200 nM	60°C	10 sec	20 sec	20 sec	530 nm/555 nm
SPUD	600 nM	200 nM	56°C	10 sec	10 sec	20 sec	470 nm/510 nm

### Inhibition assessment method

In all experiments the appropriate spiked molecule was included at ~1000 copies/reaction. Inhibition was assessed by comparing the Ct of the control reaction to which RNAse/DNAse-free water (Sigma, Cambridge, UK) had been added with the Ct of the reaction to which the potential inhibitor had been added. Inhibition was expressed as increase in Ct or as reduction in reported copy number.

### Inhibitory samples

DNA extracts from 19 urine samples were used to investigate inhibition of the SPUD and mtLSU133 PCR reactions. Unextracted urine also obtained from a single healthy male volunteer was used to investigate inhibition of all six PCR reactions. Unextracted urine was added directly to the PCR reactions to comprise 4%, 6.6%, 8%, 10%, 13.3%, 20% or 40% of the total reaction volume. In addition EDTA was used to investigate whether limiting free Mg^2+ ^would have a similar inhibitory effect on the respective reactions as unextracted urine. PCR reactions were performed as described above with the addition of 1.0, 1.25, 1.5, 2.0, 2.5 or 4.0 mM EDTA to each reaction. All experimentally inhibited reactions were performed in triplicate.

### Statistical analysis and amplicon characterisation

Statistical comparisons were made using the t test. Primer and amplicon sequences were assessed for size, GC content and secondary structure to establish if there were any sequence characteristics predictive of the degree of susceptibility to inhibition. Amplicon secondary structure was analysed using Mfold [15]. Primers were further assessed for Tm, 3' end stability, enthalpy, entropy and free energy, calculated by the nearest neighbour method [16] using NetPrimer software (Premier Biosoft International).

## Results

### Inhibition by urine extracts

**Figure 1:**
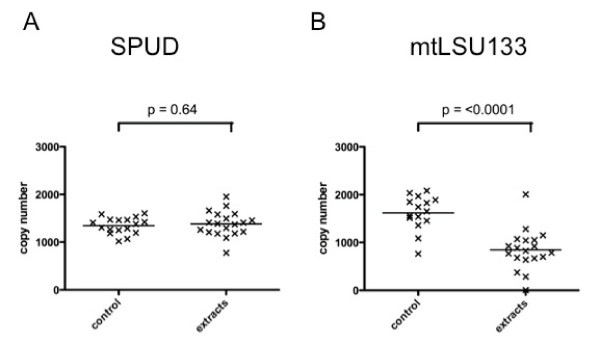
Effect of urine extracts on copy number measured by A) SPUD and B)  mtLSU133 PCR reactions.

15 of the 19 urine extracts caused an increase in Ct of > 0.5 cycle with the mtLSU133 reaction whereas only one of the extracts caused such a Ct increase with the SPUD reaction. There was no significant difference between the SPUD control reactions and the SPUD reactions to which urine extracts had been added (Figure 1A). However, there was a 1.9 fold decrease (p = < 0.0001, 95% confidence intervals 0.6 fold to 3.3 fold decrease) in the average copy number of the mtLSU133 extract reactions when compared with the mtLSU133 control reactions (Figure 1B). Thus the mtLSU133 PCR appeared to be susceptible to inhibition by urine extracts whilst the SPUD PCR reaction was not.

### Inhibition by unextracted urine

**Figure 2:**
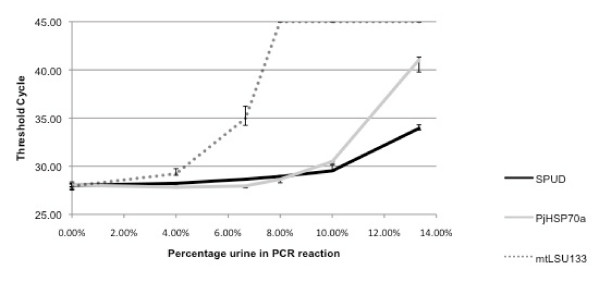
**Effect of urine concentration on threshold cycle (Ct) for three different real-time PCR reactions**. n.b. Data plotted as median ± highest and lowest (triplicate reaction), failure of reaction to amplify is represented graphically by a Ct of 45.

To further investigate PCR reaction susceptibility to inhibition, different quantities of unextracted urine were used to simulate inhibition. When three different PCR reactions (mtLSU133, SPUD and PjHSP70) were investigated there was always a positive correlation between the percentage of urine and the Ct value. Unextracted urine comprising 20% and 40% of the reaction volume totally inhibited all PCRs. When lower percentages of urine were used, the degree of inhibition was found to be reaction specific. The SPUD reaction was least affected by inhibition, the mtLSU133 reaction was most affected, with the PjHSP70 reaction intermediate (Figure 2).

**Figure 3:**
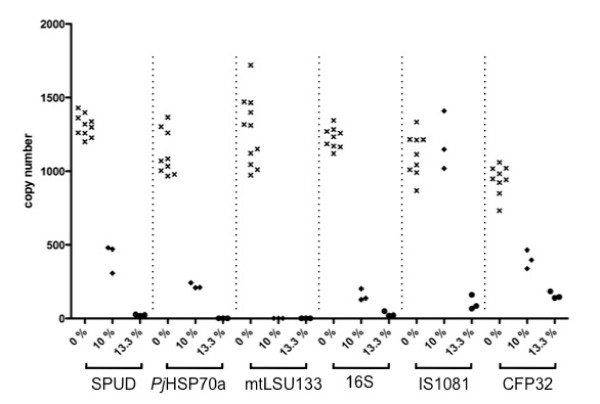
**Effect of adding 0%, 10% or 13.3% urine on copy number for six different real-time PCR reactions**. Failure of reaction to amplify is represented graphically as zero copies.

The effect of unextracted urine at 10% and 13.3% of the reaction volume was also investigated in three additional PCR reactions, 16S, CFP32 and IS1081. The results are summarised together with those generated by mtLSU133, SPUD and PjHSP70 PCRs, in Figure 3. Different degrees of susceptibility to inhibition were displayed by each of the different PCR reactions. This was most clearly illustrated by the fact that 10% urine had no inhibitory effect on the IS1081 reaction whilst completely inhibiting the mtLSU133 reaction.

### Inhibition by ethylenediaminetetraacetic acid (EDTA)

**Figure 4:**
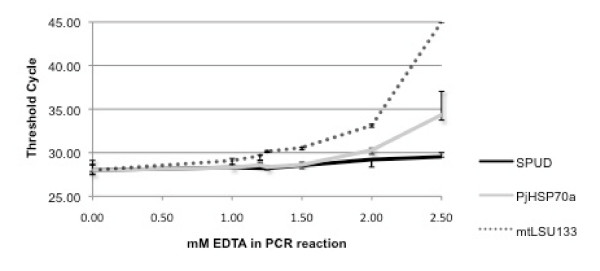
**The effect of EDTA concentration on different qPCR reactions** n.b.  Data plotted as median ± highest and lowest (triplicate reaction), failure of reaction to amplify is represented on graph by a Ct of 45. The control reactions without addition of EDTA gave a Ct value of 28 cycles.

To establish whether lowering the free Mg^2+ ^would have a similar differential inhibitory effect, the SPUD, PjHSP70a and mtLSU133 reactions were performed in the presence of different concentrations of EDTA. 4 mM EDTA completely inhibited all reactions (data not shown). Lower concentrations of EDTA produced varying degrees of inhibition that, once again, were reaction specific. The order of susceptibility to inhibition (mtLSU133 > PjHSP70a > SPUD) was the same as that observed with unextracted urine (Figure 4).

### Effect of inhibition on curve gradient and endpoint fluorescence

**Figure 5:**
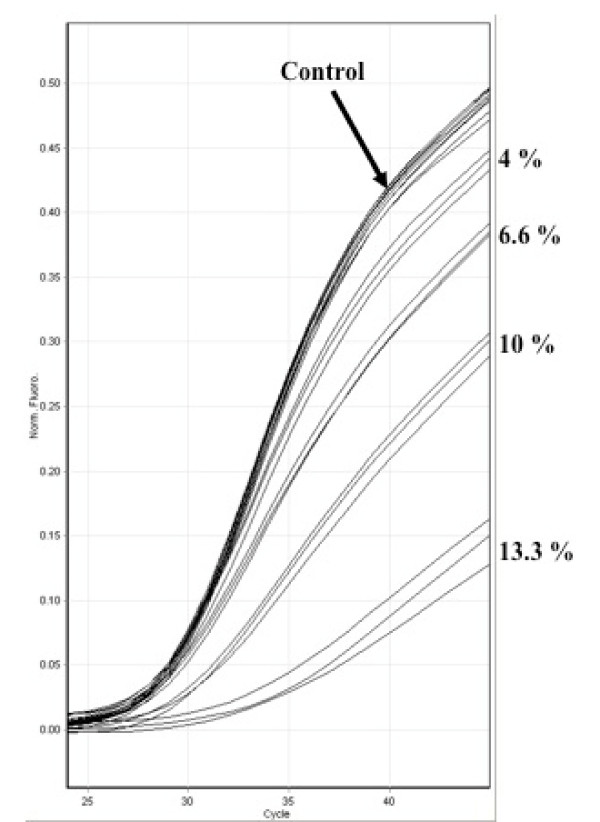
**The effect of adding 4 %, 6.6 %, 10 % and 13.3 % urine on the amplification curve gradient and endpoint fluorescence of the SPUD PCR reaction.** Background normalised data plotted using Rotor-gene 6000 series software (Corbett Research, Cambridge, UK).

Inhibition associated with urine extracts, unextracted urine or EDTA always resulted in a reduction in the steepness of the amplification curve gradient and an associated reduced endpoint fluorescence that was inversely correlated with Ct (Figure 5).

### Amplicon characteristics and susceptibility to inhibition

There was no consistent correlation across the six PCR reactions between susceptibility to inhibition and any of the primer or amplicon characteristics analysed including size, Tm, GC content, secondary structure, 3' end stability, enthalpy, entropy and free energy (Table 3). Despite the lack of a consistent or statistically significant correlation across all six reactions, it was noted that the PCR with the greatest susceptibility to inhibition (mtLSU133) generated the amplicon with the lowest GC content (32.3%) and had the lowest primer Tms, whereas the PCR that was least susceptible to inhibition (IS1081) generated the amplicon with the highest GC content (67.4%) and had the highest primer Tms. However, this trend was not maintained for PCRs with intermediate susceptibilities to inhibition.

**Table 3:** Primer and amplicon characteristics

	Primer										Amplicon	
Assay	F/R	Length bp	Mw	Tm°C	%GC	dG free energy	3' end stability	dH enthalpy of the oligo	Ds entropy of the oligo	5' end dG	Size bp	% GC
*Pj*HSP70a	F	23	6949	59.43	43.48	-37.01	-8.51	-170.1	-0.45	-8.13	108	47.2
	R	20	6053	57.87	55	-34.14	-6.47	-153.4	-0.4	-7.58		
mtLSU133	F	20	6166	**54.91**	50	-32.16	-9.31	-146.8	-0.38	-8.03	133	**32.2**
	R	19	5777	**54.46**	52.63	-31.51	-8.53	-142.9	-0.37	-6.24		
CFP32	F	20	6233	57.32	50	-34.59	-8.57	-158.5	-0.42	-6.72	63	58.7
	R	18	5547	55.49	61.11	-30.83	-7.58	-134.5	-0.35	-9.6		
IS1081	F	22	6638	**69.72**	63.64	-41.97	-13.43	-174.5	-0.44	-8.29	135	**67.4**
	R	21	6496	**67.86**	61.9	-40.00	-8.48	-166.8	-0.43	-9.5		
16S MTb	F	20	6175	56.81	50	-33.71	-7.58	-153.3	-0.4	-6.84	71	54.9
	R	20	6062	55.73	55	-32.04	-5.59	-143.4	-0.37	-8.27		
SPUD	F	23	6999	62.07	43.48	-39.56	-8.2	-180.2	-0.47	-6.83	101	42.6
	R	23	6928	61.77	43.48	-37.62	-8.32	-167.4	-0.44	-6.71		

N.B. Bold numbers correspond to factors potentially associated with susceptibility to inhibition.

## Discussion

Co-purification of inhibitors of PCR during nucleic acid extraction is a well recognised phenomenon [17,2-4] that can be caused by numerous substances [18,19]. The present study demonstrates that these inhibitors can have different effects on different PCR reactions, and that these differential effects can be concentration dependent. The latter point is particularly relevant as the actual concentration of a co-purified inhibitor is usually unknown.

The effect of reaction specific inhibition can be relatively subtle although statistically significant, as demonstrated here using urine extracts (Figure 1). In contrast, more dramatic effects may be seen at higher concentrations of inhibitor, as illustrated in Figures 2 &3. It is possible for one PCR reaction to be unaffected by a potential inhibitor whilst another is completely suppressed. If two different PCR reactions are to be compared, or one is to be used as a reference reaction for the other, as in the 'normalisation' procedure commonly used in quantitative gene expression studies [11], it is important that the two reactions are affected by potential inhibitors to the same extent; we describe this as *inhibition compatibility*. Recognition of the importance of assessing inhibition compatibility should contribute to reducing error and increasing accuracy in both gene expression studies and PCR-based molecular diagnostics. Inhibition incompatibility is likely to have a major effect on recent developments in the field, like multiplexed tandem PCR [20] and PCR arrays [21], that aim to allow many PCR reactions to be performed on a single sample.

Susceptibility of a PCR reaction to inhibitors is an important factor influencing the robustness of an assay that should be considered during experimental design. If different reactions are to be compared then they need to be of similar robustness, i.e. to be inhibition compatible. These observations lead to the question of how inhibition compatibility can be measured. An initial approach would be to perform inhibition assessment for the relevant PCR reactions in the presence of different concentrations of EDTA, as described in the present study. This is a simple strategy but it assumes that all potential inhibitors will have the same effect on the respective reactions as EDTA. The results obtained here using EDTA suggest that a PCR reaction that is more susceptible to inhibition by depletion of free Mg^2+ ^may also be more susceptible to inhibition by urine or urine extracts. This implies that the inhibitors present in urine might also be acting by depletion of free Mg^2+^, although this remains uncertain. Perhaps a more thorough approach would be to assess the relevant PCR reactions for inhibition using a range of concentrations of several well known inhibitors such as heparin and ethanol in addition to EDTA. Ideally, nucleic acid extracts from the sample types of interest (assuming they are known not to contain the PCR target) should also be tested for their inhibitory potential with the relevant PCR reactions but this may not always be feasible.

Ideally, it would be possible to design PCR reactions to be inhibition compatible and to minimise inhibition susceptibility. Unfortunately, the findings of this study have not revealed any primer, or amplicon parameter that is reliably and consistently associated with susceptibility to inhibition, although we can tentatively suggest that amplicon GC content and primer Tm may be significant factors. While it is unlikely that there is a single simple factor that can be manipulated in experimental design to ensure inhibitor compatibility there are a number of general measures that can be taken to minimise the problem. These measures include the careful selection of type of thermostable DNA polymerase [22], reduction in the amount of DNA template added to the reaction and the use of certain additives such as bovine serum albumin, which provide some resistance to inhibitors that may be present in blood [23]. Increasing the denaturation time can also reduce the susceptibility to inhibition in certain cases (data not shown).

## Conclusion

The findings of this study tackle an area that is frequently overlooked when conducting PCR. Whilst we acknowledge that our experiments use an unusual approach, as unextracted urine is not routinely used in PCR reactions (although unextracted urine has recently been used for viral genome detection [24]), the results illustrate an important principle. Users should be aware that co-purified inhibitors in nucleic acid extracts may not affect all PCR reactions equally and this fact must be taken into account when considering sample choice, experimental design and data interpretation.

## Abbreviations

Ct: threshold cycle

## Authors' contributions

JFH conceived and designed the study and analysed and interpreted the data. JFH also wrote the manuscript with the assistance of JAG, RFM and AZ. TN and CG conducted the laboratory experimentation and assisted in data analysis. SDMJ conducted the donor sampling and acquisition of consent and reviewed the manuscript. All authors read and approved the final manuscript.
